# Effective Management of a Giant Deforming Pleomorphic Adenoma With Airway Displacement in a 93-Year-Old Patient: A Case Report

**DOI:** 10.7759/cureus.68175

**Published:** 2024-08-30

**Authors:** Julio A Palomino-Payan, Jessica Guillen-Valles, Daniel A Meza-Martinez, Fernanda Urias, Luis D Montes de Oca-Gordoa

**Affiliations:** 1 General Surgery, Instituto Mexicano del Seguro Social, Hospital General de Zona No. 33, Monterrey, MEX; 2 Oncology, Instituto Mexicano de Seguros Social (IMSS) Unidad Medica de Alta Especialidad No. 25, Monterrey, MEX; 3 Pathology, Instituto Mexicano de Seguros Social (IMSS) Unidad Medica de Alta Especialidad No. 25, Monterrey, MEX

**Keywords:** head & neck pathology, cervical masses, neck neoplasm, submandibular salivary gland, head and neck oncology, salivary gland tumor, benign pleomorphic adenoma

## Abstract

Benign salivary gland tumors are a rare and diverse group of neoplasms with significant variations in their site of origin, histological features, and biological behavior. This report describes the case of a 93-year-old woman with a markedly enlarged left cervical mass. Physical inspection uncovered a tumor of approximately 32 x 30 cm, featuring necrotic and ulcerated areas. The neoplasm, diagnosed as a pleomorphic adenoma (PA) through prior biopsies, had been growing gradually over fifteen years, with delayed surgical intervention due to concerns about her age and the tumor’s size. Preoperative contrast-enhanced CT imaging showed a large left-sided cervical mass in close proximity to the airway, but without displacement or infiltration into major structures. An elective surgical approach was undertaken, involving complete resection of the giant PA, confirmed by histopathological evaluation. During the first month of postoperative follow-up, the patient experienced partial facial nerve paralysis but showed no evidence of tumor recurrence. Despite the tumor's considerable size, proximity to the airway, and the patient's advanced age, curative surgical intervention proved feasible. This case underscores that, with meticulous preoperative planning and careful surgical execution, age should not be a contraindication for surgery.

## Introduction

Pleomorphic adenomas (PAs) are the most common type of salivary gland tumor, accounting for up to two-thirds of all cases [1]. These neoplasms have a dual origin, involving both epithelial and myoepithelial components. Typically, PAs are painless, slow-growing, and usually well-circumscribed. Although there is some evidence linking the development of PAs to prior head and neck irradiation, most cases occur sporadically [1,2].

While the parotid gland is the most commonly affected site, PAs can also occur in other major salivary glands, such as the submandibular gland, as well as in minor salivary glands. In cases where the submandibular gland is affected, PAs are usually less than 6 cm at their greatest dimension and are characterized by a slow-growing, asymptomatic, unilateral firm mass that can enlarge over time if left untreated. In the minor salivary glands, PAs predominantly occur in the soft and hard palate due to the higher concentration of salivary glands in these areas. They may manifest as firm or rubbery submucosal masses, either with or without ulceration [3]. The risk of malignant transformation in these tumors is generally low; however, it increases with the duration the lesion remains untreated. Needle biopsy is considered the most reliable diagnostic method for PAs, while the standard treatment is wide surgical excision with negative margins. Local recurrence is rare but can occur, particularly in cases with significant tumor size or incomplete resection [4].

In this case, we present a patient who, despite the considerable neoplasm size, its proximity to the airway, and advanced age, underwent curative surgical resection. This case underscores the effectiveness and feasibility of surgical removal for considerably large PAs, demonstrating that neither advanced age, submandibular origin, nor significant tumor size should be considered absolute barriers to surgery.

## Case presentation

A 93-year-old female presented at our outpatient clinic with a large tumor in the left submandibular region, initially detected during her mid-fifties. She had previously undergone a core biopsy a year prior, which confirmed a diagnosis of PAs in the left submandibular gland. Her past medical history was unremarkable.

The patient reported that the tumor had been gradually growing since she first noticed it, but over the past fifteen years, the growth had accelerated significantly. Despite noticing the accelerated growth, she sought medical treatment without experiencing any general symptoms, dyspnea, or dysphagia.

On physical examination, the tumor extended throughout the entire left anterolateral region of the neck, measuring approximately 32 x 30 cm. It exhibited a solid consistency, discrete mobility, tenderness to palpation, and areas of necrosis and ulceration in its lower third, along with remnants of bleeding. No other significant medical or surgical findings were noted.

**Figure 1:**
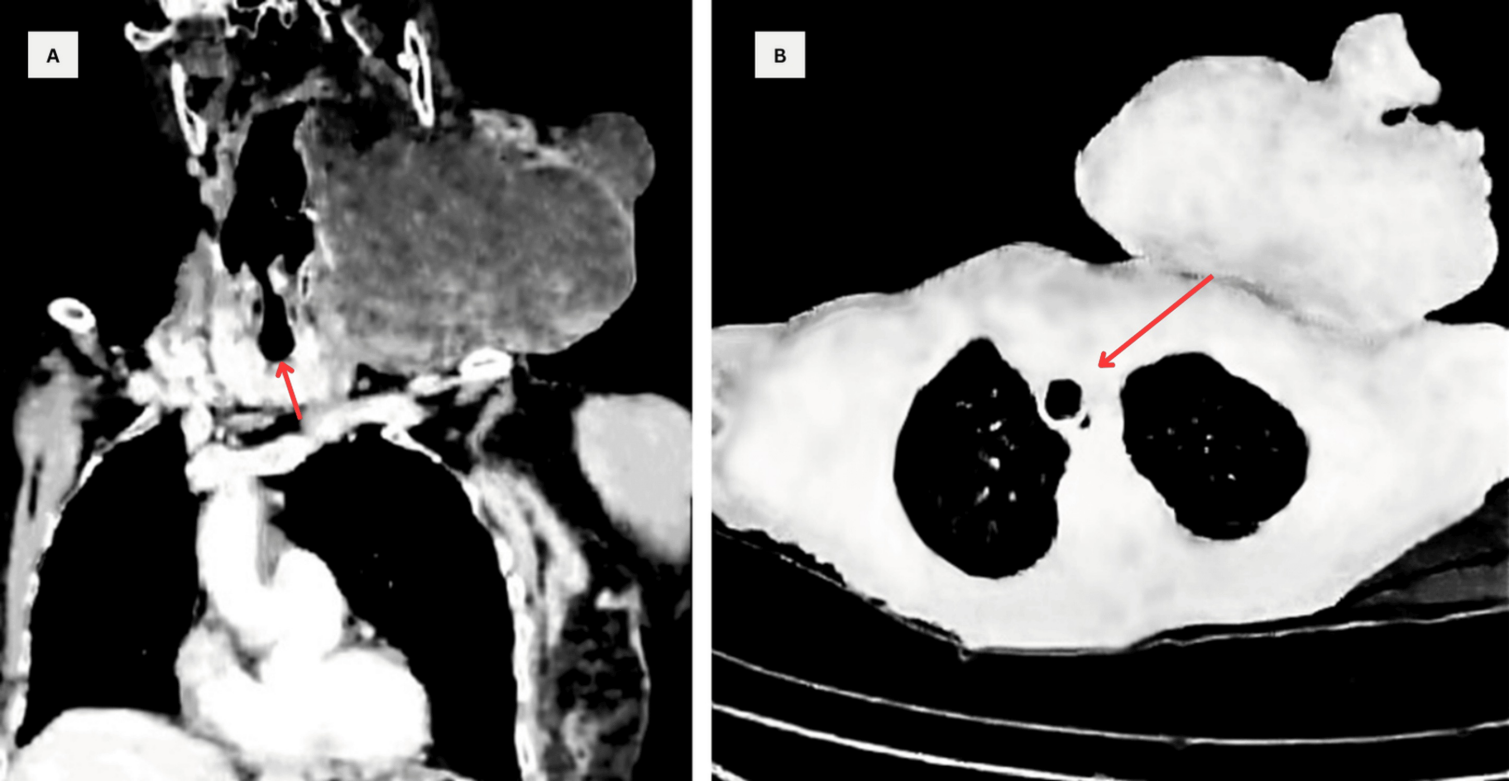
Contrast CT Images A: Coronal image showing a clearly demarcated, heterogeneous left cervical mass measuring approximately 30 x 25 cm, likely originating from the submandibular region, involving adjacent structures with no significant airway obstruction (red arrow). B: Axial images demonstrating tracheal deviation with a patent airway and no signs of obstruction (red arrow).

The patient expressed concerns about surgical intervention due to her age and the substantial tumor volume, leading her to decline treatment when first assessed a year ago. A preoperative CT with contrast revealed a large left-sided cervical tumor measuring 30 x 25 cm with airway displacement (Figure 1).

The patient was admitted for completion of laboratory tests and further evaluation, including radiation oncology and medical oncology consultations. She remained asymptomatic, required no oxygen, and maintained an enteral diet. Subsequent evaluations by the surgical oncology team determined the necessity of primary operative treatment.

**Figure 2:**
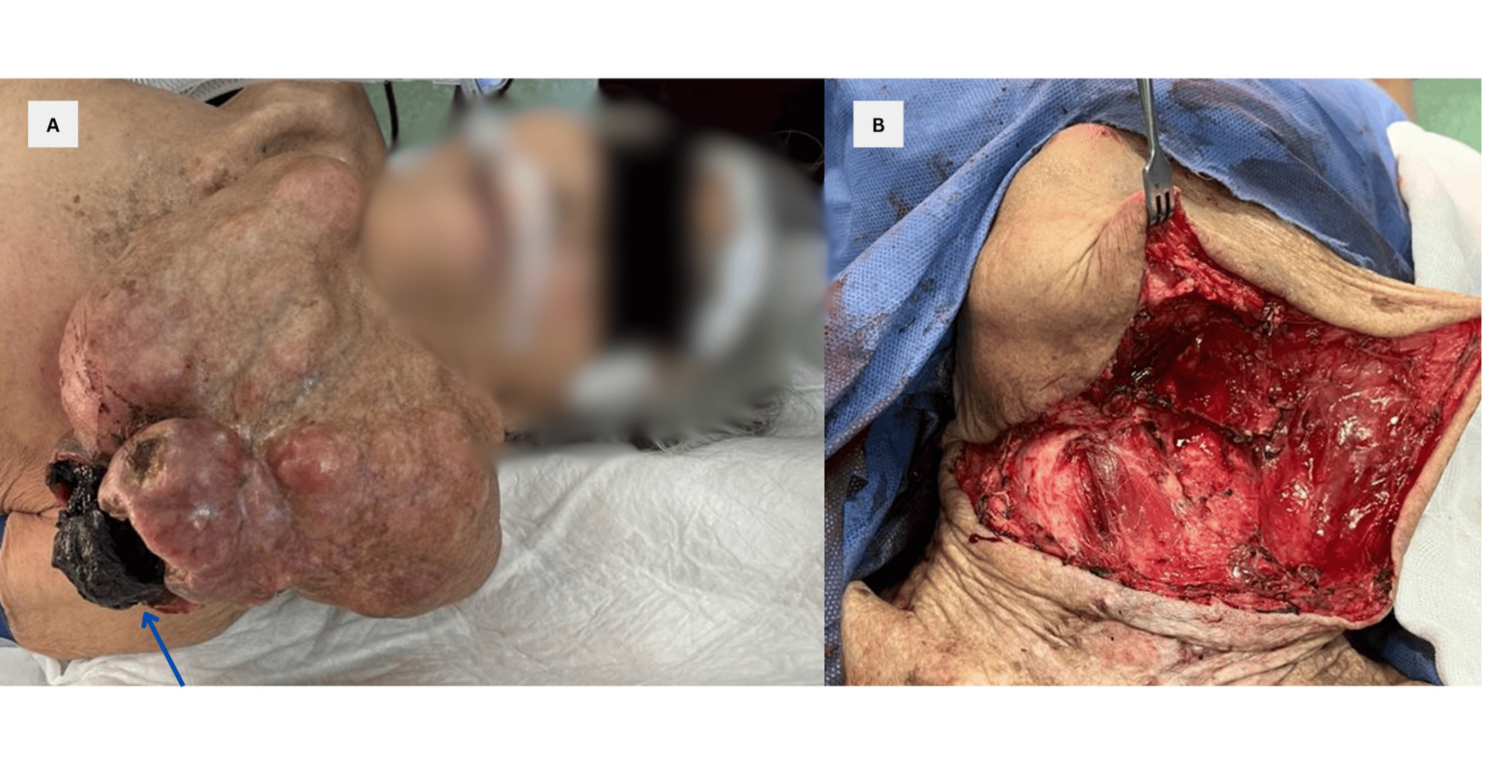
Perioperative findings A: Preoperative examination reveals a multilobulated giant mass located in the left anterolateral neck region, extending along the inferior border of the left mandible. An area of ulceration and necrosis measuring 8 x 5 cm is identified on the lower aspect of the tumor (blue arrow). B: Intraoperative visualization of the complete tumor resection through cephalic cutaneous flap exposure.

Surgery was planned with the primary goal of achieving wide resection. The procedure was performed under general anesthesia following informed consent. The patient was placed in a supine position with neck hyperextension. The surgical approach began with a peritumoral skin incision, followed by dissection through the layers to reach the platysma muscle. Flaps were then raised in both cephalic and caudal directions (Figure 2).

During dissection, the facial nerve was carefully exposed, with particular attention to the marginal mandibular branch. Due to excessive tumor growth and significant anatomical distortion, the nerve branch was not correctly identified, complicating the dissection process and increasing the risk of nerve injury. Despite these challenges, the surgeon proceeded to identify and ligate the salivary duct and vascular pedicle.

**Figure 3:**
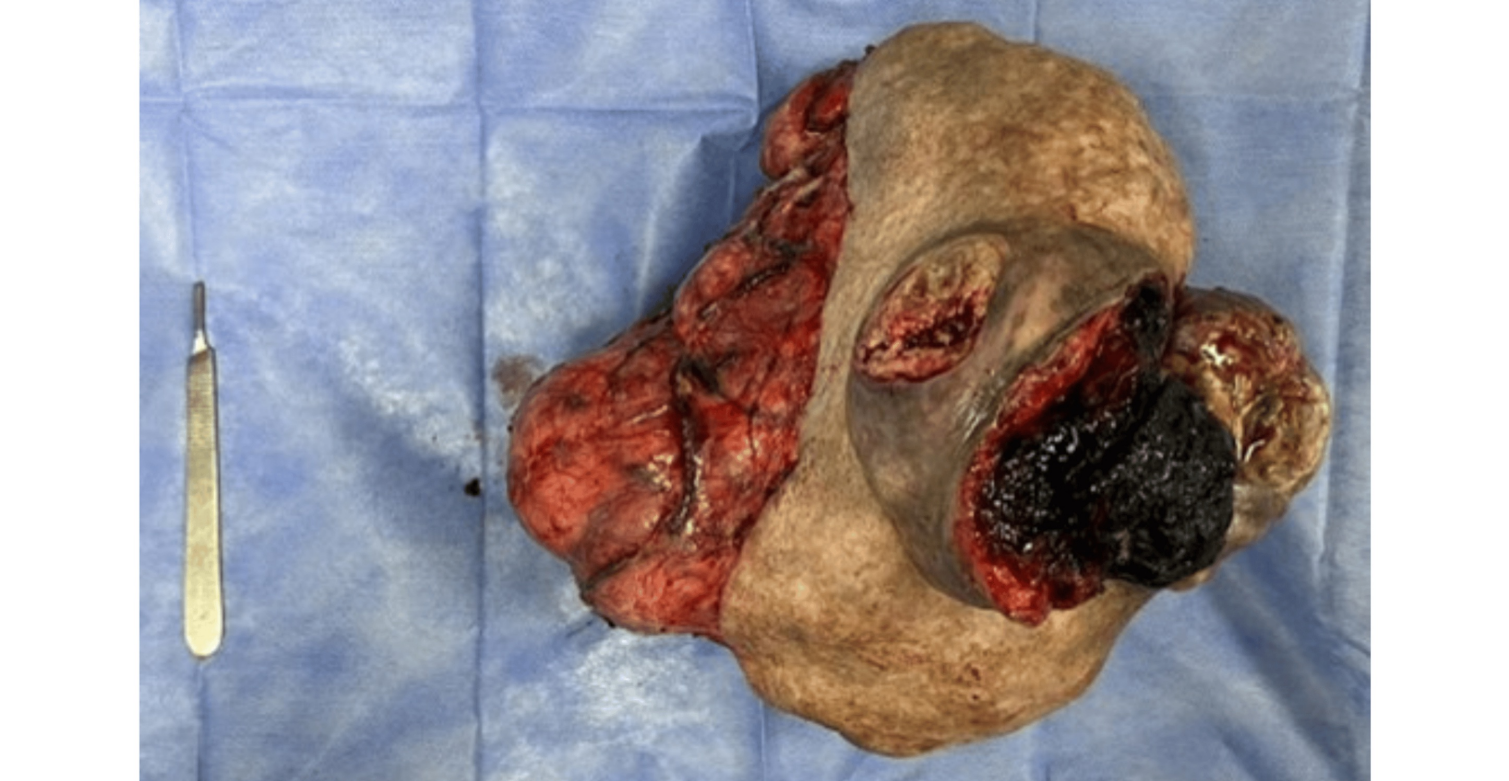
Surgical specimen Complete tumor excision revealed a multilobulated neoplasm measuring 30 x 26 x 16 cm, with a firm consistency and necrotic skin ulcers on the anterior surface.

Given the distorted anatomy, the next step involved carefully isolating the tumor from surrounding tissues to ensure complete excision. The surgeon meticulously navigated the altered tissue planes to remove the tumor while preserving nerve and tissue integrity. The neoplasm was then completely excised, and a closed drainage system was placed to prevent fluid accumulation. After completion, histopathological examination followed. In gross examination, the surgical specimen was multilobulated, measuring 30 x 26 x 16 cm, and firm in consistency, with necrotic skin ulcers on the anterior surface. No macroscopic evidence of regional invasion or regional lymphadenopathy suggestive of metastasis was observed (Figure 3).

The patient remained under observation to manage any potential complications. The only complication identified was a left facial nerve injury. After the procedure, the patient was closely monitored. The 24-hour postoperative review showed no signs of bleeding through the incision or closed drainage. The surgical flap was in good condition with adequate coloration, showing no signs of ischemia or perfusion abnormalities. The patient remained under observation to manage any potential complications.

**Figure 4:**
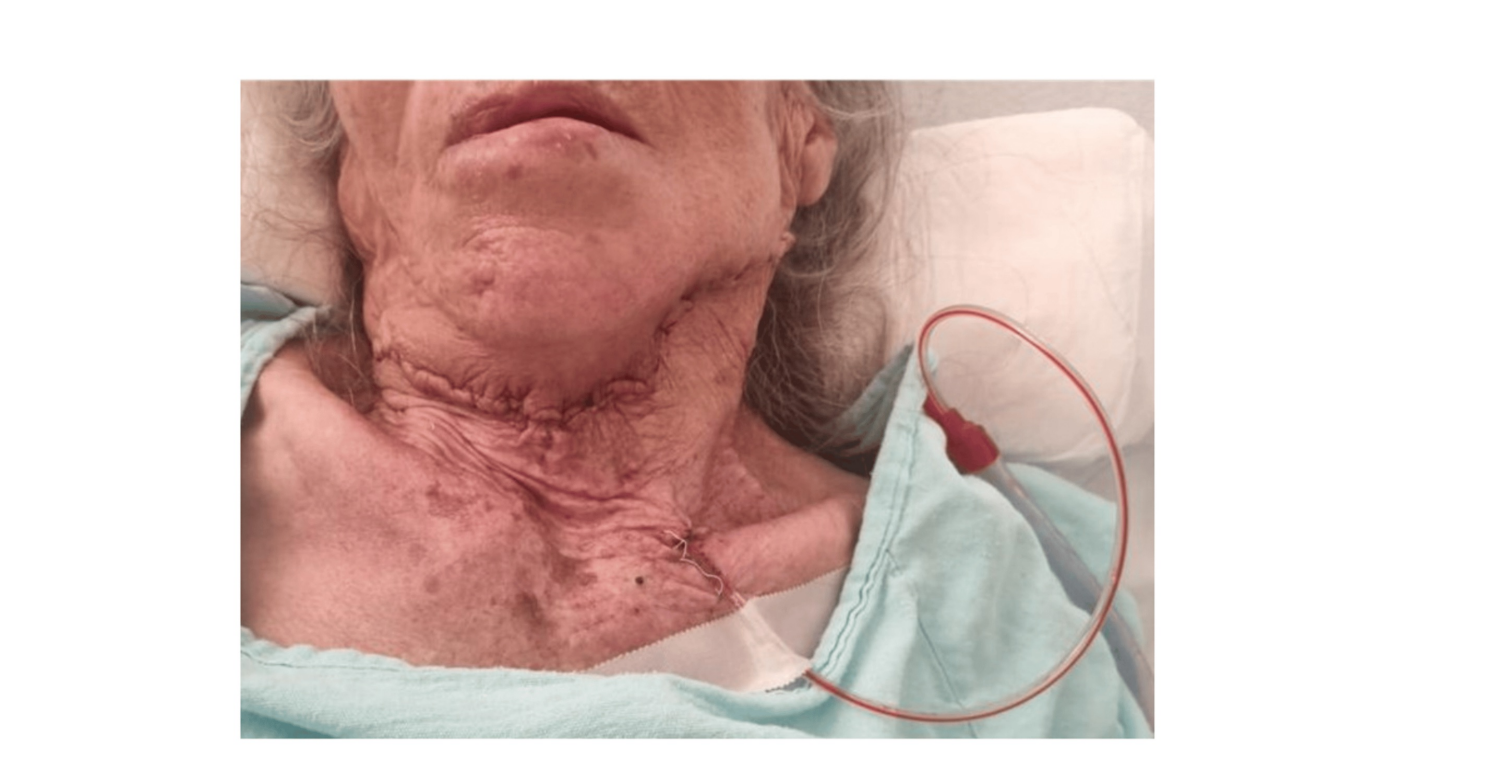
Postoperative evaluation Physical examination 24 hours post-surgery revealed a well-colored skin flap with no signs of bleeding, ischemia, or necrosis. The closed drainage system was functional, with no evidence of fluid accumulation or active bleeding. Findings suggestive of injury to the left marginal mandibular branch of the facial nerve were noted during the physical exam.

Postoperative examination revealed signs of partial left facial nerve injury, characterized by slight facial asymmetry, inability to depress the left lower lip, and ptosis of the left oral commissure, indicating damage to the left marginal mandibular branch of the facial nerve, compromising the depressor anguli oris and depressor labii inferioris muscles (Figure 4).

**Figure 5:**
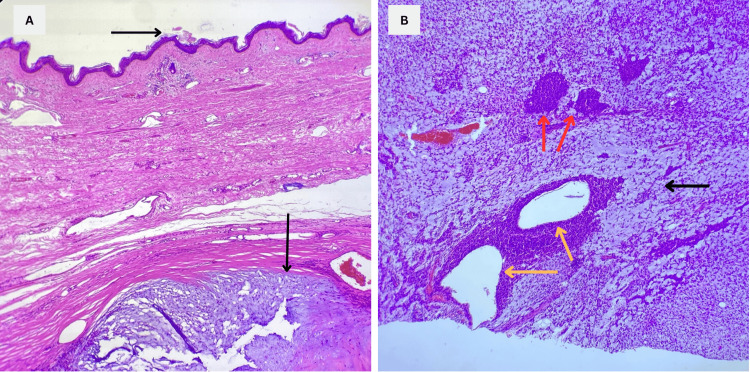
Microscopic findings A: Histopathological image showing the neoplasm with the overlying epidermal layer of the skin (arrow pointing rightward). The deep dermis exhibits replacement of the dermal tissue by neoplastic tissue which is consistent with the PA (arrow pointing downward). B: Histopathology reveals the three elements of the pleomorphic adenoma: cyst-forming epithelial ducts (yellow arrows), dispersed myoepithelial cells (red arrows), and the stromal component, which appears as the tumor's myxoid matrix with a characteristic light blue background (black arrow). PA: Pleomorphic adenoma

Once the histopathology results were available, the diagnosis of the PA was reiterated, aligning with previous biopsy findings in her medical history and showing no evidence of malignancy, as demonstrated by the lack of basal membrane infiltration and the typical features of the PA (Figure 5).

**Figure 6:**
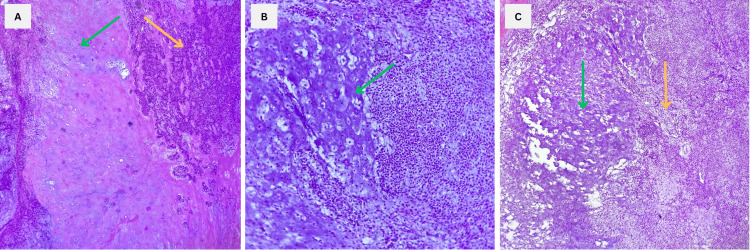
Histopathological images showing findings consistent with the PA A: 10x. Image showing the hallmark findings of PA: chondromyxoid stroma (green arrow) and myoepithelial cells (yellow arrow). B: 10x. Representative image showing the presence of cartilage within the chondromyxoid stromal component C: 40x. Image highlighting the myoepithelial component (yellow arrow) alongside the chondromyxoid stromal component, which exhibits cartilage and mucin formation (green arrow). PA: Pleomorphic adenoma

Hematoxylin and eosin (H&E) staining demonstrated a fibrous capsule surrounding heterogeneous zones of epithelial, myxoid, and ductal elements, exhibiting the classic features of the PA, including myoepithelial and ductal cells (Figure 6).

The patient was discharged on the third postoperative day after demonstrating tolerance to an oral diet, ambulating independently, and having normal bowel movements. She was discharged with the drainage system and scheduled for a follow-up appointment one week later for reevaluation. The patient returned for drainage removal, and no complications were observed. However, she did not attend subsequent follow-up appointments, and her subsequent outcome remains unknown.

## Discussion

The PA, also referred to as a benign mixed tumor, is the most common type of salivary gland neoplasm, accounting for up to 75% of all cases. This tumor is notable for its potential to recur locally and its risk of transforming into a malignant form known as carcinoma ex-pleomorphic adenoma. Although it predominantly arises in the parotid gland, PAs can also develop in the submandibular gland and minor salivary glands dispersed throughout the mouth's submucosa and the upper aerodigestive tract [5]. 

The incidence of PAs in the elderly population is relatively low. However, when they do occur, their long-standing presence and slow growth rate often lead to a significant tumor size by the time of diagnosis [6]. This case is particularly notable due to the patient's advanced age, in addition to the tumor's dimensions. In this instance, she had a markedly enlarged left cervical mass that had been growing over several decades, and its substantial size inevitably led to significant mechanical symptoms, including discomfort, aesthetic concerns, and minor functional impairments due to the displacement of surrounding structures.

Accurate diagnosis of PAs involves a combination of clinical evaluation, imaging studies, and histopathological examination. Fine needle aspiration biopsy is a reliable method for initial diagnosis, while imaging studies such as contrast-enhanced CT scans help delineate the tumor's extent and its relationship with surrounding structures [4]. Both needle biopsy and CT imaging confirmed the diagnosis in this case, showing characteristic findings of PA. Despite the diagnosis having been established through biopsy in the past, it is noteworthy that the tumor never underwent malignant transformation.

The main treatment for PAs is surgical excision, aiming for complete removal of the tumor with clear margins to minimize the risk of recurrence. Due to the encapsulated nature of these neoplasms, a meticulous surgical technique is essential to prevent rupture and spillage of tumor cells, which could lead to recurrence [3,4,6]. Surgical intervention in elderly patients requires a careful risk-benefit assessment. The potential benefits of tumor removal must be weighed against the risks of anesthesia and surgical complications. Anesthetic management in elderly patients is complex due to age-related physiological changes and comorbid conditions. Preoperative optimization and careful intraoperative monitoring are essential to minimize risks [4,6,7]. The decision to proceed with optimal treatment required a careful risk-benefit assessment. The potential benefits of tumor removal were weighed against the risks of anesthesia and surgical complications, particularly the risk of airway compromise. The patient's favorable health status and the non-malignant nature of the tumor supported the decision to proceed with surgery. Despite the complexities posed by advanced age, appropriate surgical intervention resulted in favorable outcomes.

The primary complication associated with PA resection is temporary or permanent facial nerve paralysis [8]. Postoperatively, the patient developed partial facial nerve paralysis, a known risk given the tumor’s size and location. Furthermore, the patient’s skin graft healed well, with no other evidence of complications. This favorable outcome highlights the importance of meticulous surgical technique and comprehensive perioperative management in achieving successful results, even in high-risk, elderly patients.

Long-term monitoring is essential for PA cases to detect potential recurrences, especially after incomplete resections [1]. In this instance, despite a brief follow-up period, no tumor recurrence was observed, indicating a positive prognosis. Nevertheless, the limited follow-up duration means that long-term outcomes remain uncertain. However, this case demonstrates that, with proper work-up and surgical technique, favorable outcomes are achievable even for elderly patients with giant PAs.

## Conclusions

This case report illustrates the successful management of a large PA in a 93-year-old patient, demonstrating that even extensive, long-standing tumors can remain benign. This report highlights the critical role of the meticulous surgical technique in avoiding facial nerve damage, which is particularly challenging in such cases, and in preventing recurrence given the tumor's well-circumscribed nature. Postoperatively, the patient developed partial facial nerve paralysis, a recognized risk due to the tumor’s size and location, but had no other complications, including successful skin graft healing. Remarkably, despite the patient's advanced age, there were no airway complications or perioperative management issues. This outcome underscores the effectiveness of tailored surgical approaches and comprehensive perioperative care, showing that elderly patients with large tumors can achieve favorable results. Continued monitoring is necessary to detect any potential recurrences early, ensuring ongoing patient health and favorable long-term outcomes.
